# Enhancing human and animal health data integration and informed actions for pandemic preparedness at the primary healthcare level: a multisectoral conceptual framework

**DOI:** 10.7189/jogh.15.03018

**Published:** 2025-04-02

**Authors:** Bach Xuan Tran, Ha Ngoc Vu, David B Duong, Laurent Boyer, Tran Hoang Long, Duy Cao Nguyen, Shenglan Tang

**Affiliations:** 1 Faculty of Public Health, VNU University of Medicine and Pharmacy, Vietnam National University, Hanoi, Vietnam; 2 Bloomberg School of Public Health, Johns Hopkins University, Maryland, USA; 3 Division of Global Health Equity, Brigham and Women’s Hospital, Boston, Massachusetts, USA; 4 Harvard Medical School Center for Primary Care, Boston, Massachusetts, USA; 5 CEReSS-Health Services Research and Quality of Life Center, Aix-Marseille University, France; 5 Dong Nai Technology Universitxy, Dong Nai, Vietnam; 7 Aston University, Birmingham, UK; 8 VNU University of Economics and Business, Vietnam National University, Hanoi, Vietnam; 9 Institute for Global Health Innovations, Duy Tan University, Vietnam; 10 Duke Global Health Institute, Duke University, North Carolina, USA

## Abstract

A key priority for strengthening global health capacity for pandemic response is rapid risk assessment for timely, context-specific decision-making. However, integrating human and animal health data for preparedness remains a challenge, especially at the primary healthcare (PHC) level. Here we review Vietnam’s pandemic response and propose a conceptual framework for improving data integration across sectors in low- and middle-income countries. Despite the country’s progress in health information systems and telehealth, disparities in data use and coordination between human and animal health sectors hindered effective responses. Existing mechanisms between healthcare and veterinary professionals lack integrated data-sharing, delaying risk communication and crisis management, particularly in rural areas with limited IT access and infrastructure. The proposed model includes five components: data interoperability with standardised indicators for real-time synthesis; robust digital health infrastructure and telehealth expansion; capacity building in data management for health and veterinary professionals; epidemic intelligence tools for risk assessment; and evidence-driven decision-making for coordinated epidemic responses. This model offers a pathway to strengthen health systems and improve pandemic preparedness at the PHC level in Vietnam and similar settings.

One of the key priorities in strengthening global health capacity for pandemic response is the rapid risk assessment using cross-sectional evidence to inform timely decision-making in local contexts and scenarios [1,2]. However, this remains a significant challenge in many settings, but particularly in low- and middle-income countries (LMICs), where pandemic response decisions have often lacked sufficient integration into local contexts. Such countries also saw limited timely use of human and animal health data for local strategic planning and action, an issue exacerbated by the under-resourced and fragmented nature of their primary healthcare (PHC) systems [3].

Recently, Redman-White et al. [1] reviewed data-sharing strategies and introduced the Digital One Health (DOH) framework, emphasising five key dimensions: standard harmonisation, automated data capture, integrated capture, onboard analysis, and archiving and governance, aimed at improving trust, quality, efficiency, accessibility, utility, and security. However, the framework does not address its alignment with existing surveillance systems or the demand for actionable evidence at the grassroots level. Drawing from a review of Vietnam’s pandemic response, we propose a broader conceptual framework adaptable for LMICs to improve pandemic preparedness by enhancing data integration across human and animal health sectors, with a particular focus on the PHC level.

In Vietnam, efforts to strengthen health information systems (HIS) and implement telehealth were rapidly scaled up during the COVID-19 pandemic [4]. For example, telehealth services provided real-time medical consultations in remote areas, but gaps in IT literacy among local healthcare workers and limited veterinary field data reporting delayed coordinated actions. Infrastructure disparities between urban and rural areas further hindered effective integration of human and animal health data. However, significant disparities in the use and coordination of human and animal health data across sectors were evident, particularly in rural areas, where infrastructure, IT literacy, and access to data-sharing mechanisms were limited. This lack of integrated data-sharing delayed timely risk communication and crisis management, hindering an effective response at the PHC level. Although mechanisms for collaboration between healthcare and veterinary professionals exist, their implementation remains fragmented. Any collected data are reported to higher authorities, with minimal and delayed local use. This highlights the urgent need for a more cohesive, multisectoral approach to data integration and pandemic preparedness at the PHC level.

To address this gap, we propose a multilevel, interconnected framework to improve pandemic preparedness by strengthening data integration across human and animal health sectors at the PHC level (**Figure 1**). This framework builds on Vietnam’s experience, but has broader applicability for LMICs facing similar challenges following five critical components (**Box 1**).

**Figure 1:**
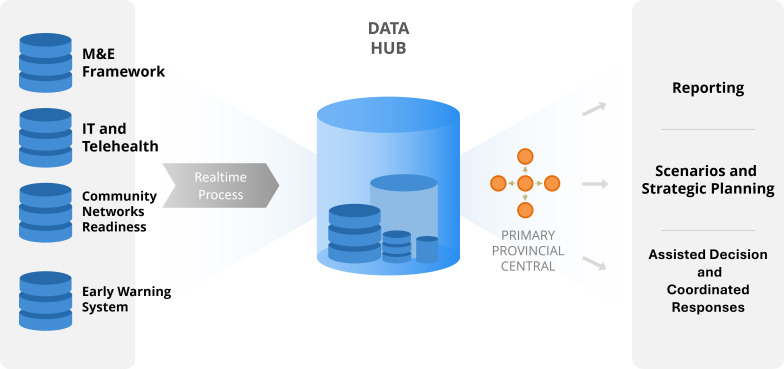
Concept of data integration and use for pandemic responses.

Box 1 **Five components of the proposed framework**

Data interoperability: the first component involves developing structured sets of indicators and standardised guidelines to enable real-time, seamless data sharing between human and animal health sectors. For instance, adopting global standards like Health Level 7 or the International Classification of Diseases, 11th Revision, can ensure that information collected in one sector is accessible, interpretable, and actionable across all relevant stakeholders, including healthcare providers and veterinary services. This would address current barriers such as incompatible data systems and inconsistent reporting practices.

Infrastructure and digital health systems: it is essential to strengthen IT infrastructure, particularly in rural and underserved areas where inadequate systems have historically hindered effective pandemic responses. During Vietnam’s COVID-19 response, mobile-based health platforms provided critical services to rural populations, but their reach was constrained by limited resources and interactions. Expanding the use of telehealth and integrating epidemic intelligence tools, such as Go.Data and District Health Information System 2, which support data collection, contact tracing, outbreak investigation, and real-time analytics, can enhance automation, improve timely decision-making, and strengthen real-time surveillance. These advancements are crucial for promoting more equitable access to care, especially in resource-limited settings, by enabling proactive and informed public health interventions.

Capacity building and IT literacy: training programmes aimed at improving IT literacy among healthcare and veterinary professionals, local authorities, and stakeholders are essential for successful data integration. Cost-effective strategies, such as mobile-based IT training and leveraging community health networks, can strengthen local-level surveillance and reporting. Capacity-building efforts should also include training on basic epidemic methods, digital tools, and data management systems to support informed decision-making during health crises.

Risk assessment and early warning systems: implementing epidemic surveillance and establishing early warning systems that utilise data from both human and animal health sectors are essential for proactive pandemic preparedness. Early warning systems involve real-time monitoring, timely data analysis, and risk communication to detect and mitigate potential threats before they escalate. Experience from Vietnam highlights significant challenges, particularly the fragmented reporting within veterinary systems for zoonotic diseases. To address these gaps, LMICs must develop integrated mechanisms at critical control points, enhance surveillance capacities, and streamline rapid risk assessment processes.

Evidence-informed strategic planning and epidemic response: data collected through interoperable systems must inform strategic planning and epidemic response efforts at all levels of health governance. Developing policies that promote data-driven, coordinated responses tailored to local contexts is critical. Vietnam’s delayed integration of PHC and veterinary services during the pandemic underscores the need for actionable local-level data to complement centralized reporting systems. For example, Vietnam’s delayed integration of PHC and veterinary services during the pandemic underscores the need for actionable local-level data to complement centralized reporting systems.

This conceptual framework, informed by the Vietnamese experience, serves as a reference for other LMICs where similar challenges exist. In resource-scarce settings, PHC systems are the first line of defence during pandemics, but are often the least equipped to manage the complexity of integrated responses required to combat zoonotic diseases and epidemics. This model highlights a key function of PHC administration, where extensive data collection and synthesis occur, but are seldom utilised effectively due to inherent resistance to cross-sector collaboration, insufficient financial incentives, and other systemic barriers. Investing in data interoperability, building digital infrastructure, and enhancing capacity across sectors will ensure that LMICs are better positioned to respond to health crises that increasingly demand cross-sectoral coordination, data-driven decision-making and shared accountability mechanisms. The time to act is now – delays in implementing integrated, multisectoral strategies could have severe consequences for public health and economic stability. This framework provides a practical roadmap for building more resilient health systems capable of managing emerging infectious diseases, particularly at the PHC level, and should be prioritised without hesitation.
